# Physical activity at age 11 years and chronic disabling fatigue at ages 13 and 16 years in a UK birth cohort

**DOI:** 10.1136/archdischild-2017-314138

**Published:** 2018-01-30

**Authors:** Simon M Collin, Tom Norris, Kevin C Deere, Russell Jago, Andy R Ness, Esther Crawley

**Affiliations:** 1 Population Health Sciences, Bristol Medical School, University of Bristol, Bristol, UK; 2 Centre for Child and Adolescent Health, University of Bristol, Bristol, UK; 3 Department of Health Sciences, Centre for Medicine, College of Medicine, Biological Sciences and Psychology, University of Leicester, Leicester, UK; 4 Musculoskeletal Research Unit, University of Bristol, Southmead Hospital, Bristol, UK; 5 Centre for Exercise, Nutrition and Health Sciences, School for Policy Studies, University of Bristol, Bristol, UK; 6 NIHR Biomedical Research Centre at the University Hospitals Bristol NHS Foundation Trust and the University of Bristol, University Hospitals Bristol Education Centre, Bristol, UK

**Keywords:** ALSPAC, chronic fatigue syndrome, adolescent health, physical activity

## Abstract

**Objective:**

To investigate associations of physical activity at age 11 years with chronic disabling fatigue (CDF) at ages 13 and 16 years.

**Design:**

Longitudinal birth cohort.

**Setting:**

South-West England.

**Participants:**

Adolescents enrolled in the Avon Longitudinal Study of Parents and Children.

**Outcomes and exposures:**

We identified adolescents who had disabling fatigue of >6 months' duration without a known cause at ages 13 and 16 years. Total and moderate-to-vigorous physical activity and sedentary time at age 11 years were measured by accelerometry over a 7-day period.

**Results:**

A total physical activity level 100 counts/min higher at age 11 years was associated with 25% lower odds of CDF at age 13 years (OR=0.75 (95% CI 0.59 to 0.95)), a 1% increase in the proportion of monitored time spent in moderate-to-vigorous activity was associated with 16% lower odds of CDF (OR=0.84 (95% CI 0.69 to 1.01)) and a 1-hour increase in sedentary time was associated with 35% higher odds of CDF (OR=1.35 (95% CI 1.02 to 1.79)). Disabling fatigue of only 3–5 months’ duration at age 13 years had weaker associations with physical activity, and CDF at age 16 years was not associated with physical activity at age 11 years.

**Conclusions:**

Children who had chronic disabling fatigue at age 13 years had lower levels of total and moderate-to-vigorous physical activity and more sedentary time 2 years previously, but this association could be explained by reverse causation.

**What is already known on this topic?**

- Lower levels of childhood physical activity were reported to be associated with increased risk of chronic fatigue syndrome (also known as ME) during adulthood.
- It is unclear whether physical activity is a risk factor for the development of chronic fatigue during adolescence.

**What this study adds?**

- Children who were less physically active and more sedentary at age 11 years had an increased risk of chronic disabling fatigue at 13 years.
- These associations were not evident at age 16 years, and could be explained by reverse causation.

## Introduction

Chronic fatigue syndrome (CFS), also known as ‘ME’ and, in the USA, as systemic exertion intolerance disease,^1^ is a debilitating disease which affects adults and children.^2 3^ UK National Institute for Health and Care Excellence guidelines state that a diagnosis of CFS/ME should be made after 3 months of persistent or recurrent fatigue which is not the result of ongoing exertion, not alleviated by rest, has resulted in a substantial reduction in activities, has no other known cause and is accompanied by one or more of 10 typical symptoms.^4^ The US Centers for Disease Control and Prevention diagnostic criteria are similar, but specify 6 months’ duration of fatigue and four or more additional symptoms.^5^ Chronic fatigue of at least 3 months’ duration affects 1%–3% of children and young people.^6–11^ Clinician-verified CFS/ME appears to be much less prevalent (0.1%–0.5%)^12–14^; this discrepancy is probably a combination of clinician diagnostic uncertainty,^15^ undiagnosed cases^16^ and inaccuracies in population-based studies.

It is unclear whether physical activity is a risk factor for paediatric CFS/ME. Lower levels of childhood physical activity were reported to be associated with increased risk of CFS/ME during adulthood,^17^ and healthy girls aged 14 years girls who were less physically active were more likely to report symptoms characteristic of CFS/ME (unrefreshing sleep, muscle pain, joint pain, headaches, tender lymph nodes, memory and concentration problems) at age 19 years.^18^ A case–control study of adolescents with infectious mononucleosis who did (cases) or did not (controls) subsequently develop CFS/ME found no differences in levels of physical activity in the 12 months before the initial infectious illness.^19^ Here we use data from the Avon Longitudinal Study of Parents and Children (ALSPAC) to investigate whether levels and patterns of physical activity at age 11 years^20^ are associated with ‘chronic disabling fatigue’ (CDF, a proxy for clinically diagnosed CFS/ME) at ages 13 and 16 years.^6 7^

## Methods

### Study cohort

ALSPAC is a population-based study that aims to investigate a wide range of influences on the health and development of children.^21^ Pregnant women residing in the former Avon Health Authority in South-West England who had an estimated date of delivery between 1 April 1991 and 31 December 1992 were invited to take part, resulting in a cohort of 14 541 pregnancies and 13 978 children alive at 12 months of age (excluding triplets and quads). The ALSPAC study website contains details of all the data that are available through a fully searchable data dictionary (www.bris.ac.uk/alspac/researchers/data-access/data-dictionary/).

### Outcomes—CDF at ages 13 and 16 years

Our methods for defining CDF at age 13 (median 13.1, IQR 13.1–13.2) and 16 (median 16.6, IQR 16.5–16.8) years have been described previously.^6 7^ In brief, mothers were asked whether their child had been feeling tired or lacking in energy over the last month (yes, no); how long the tiredness had lasted (<3 months, 3–5 months, 6 months to 5 years, >5 years); how many days their child had missed school because of tiredness; and whether the tiredness/lack of energy had prevented the child from taking part in hobbies, sport or leisure activities (not at all, only a little, quite a lot, a great deal). The questionnaires asked whether the teenager snored (never, sometimes, often), whether the mother thought the fatigue was due to the teenager playing too much sport and whether the teenager took regular medication (free text question: ‘Please indicate below any medicines your child has used in the last 12 months’). We identified adolescents reported by their mothers to have experienced fatigue lasting >6 months that was associated with absence from full-time school or that had prevented them from taking part in activities ‘quite a lot’ or ‘a great deal’. We excluded those whose mothers thought that the fatigue was caused by playing too much sport, who snored often and who had other illnesses that could cause fatigue (based on self-reported medication use). At age 16 years, children could only be classified as having CDF if they scored ≥19 (out of 33) on the Chalder Fatigue Scale.^22 23^ According to our definitions, the prevalence of CDF of >6 months’ duration at ages 13 and 16 years was 1.1% (76/6720) and 1.5% (84/5756), respectively. To reduce overlap between primary exposures and outcomes, we excluded children with >5 years’ duration of fatigue at ages 13 (10 out of 76 children) and 16 (11 out of 84 children) years. After these exclusions, 66/6720 children at age 13 years and 73/5756 children at age 16 years were classified as having CDF (of 6 months’ to 5 years’ duration). We also performed a sensitivity analysis in which we redefined our outcome at age 13 years to include only children whose disabling fatigue had been of 3–5 months’ duration (78/6720 (1.2%)).

### Primary exposure—physical activity at age 11 years

Methods for collecting physical activity data have been described previously.^20^ All ALSPAC children who attended research clinics at age 11 years (median 11.8 years, IQR 11.6–11.9 years) were asked to wear an Actigraph AM7164 2.2 accelerometer (Actigraph LLC, Fort Walton Beach, Florida, USA) for 7 days. Data are recorded as counts, which are averaged over a defined period (1 min in this study). The Actigraph has been validated in children and adolescents.^24–27^ Participants wore the accelerometer on a waist worn belt and were instructed to remove it only when showering, bathing or doing water sports. Physical activity variables were derived from the raw accelerometer counts using customised software. Data from children who had worn the accelerometer for at least 10 hours a day for at least 3 days were considered valid. Three physical activity variables were calculated—total (volume of) physical activity, moderate-to-vigorous physical activity and sedentary time.

Total physical activity was calculated as the average accelerometer counts per minute (cpm) over the full period of valid recording. To facilitate interpretation, associations with total physical activity were calculated per 100 cpm. This measure was chosen because it approximates the difference in physical activity between boys and girls and therefore provides a useful reference point.^20^ All minutes of recording with a total of ≥3500 accelerometer counts were classified as moderate-to-vigorous physical activity. The threshold of 3500 cpm was derived from a calibration study in a subsample of 246 ALSPAC children.^25^ We analysed moderate-to-vigorous physical activity as a mean daily proportion of total monitored time. This allowed us to investigate whether the volume of physical activity or (occasional) intensity of physical activity was more important in predicting risk of CDF. Sedentary time was the mean daily amount of time (hours per day) when the accelerometer recorded ≤100 cpm.

### Statistical analysis

A set of putative risk factors for paediatric CFS/ME was identified from the literature.^6 7 10 28^ To identify possible associations between our exposures and outcome, we identified confounding variables which could potentially bias any observed association.^29^ The first step in this process was to hold consultative meetings with experts in the fields related to the primary (physical activity) and other exposures (sleep, child and maternal psychopathology) and specialists in paediatric CFS/ME. Consensus from these meetings was encapsulated in the form of directed acyclic graphs (DAGs). DAGs are causal diagrams which provide a method for formalising and clarifying relationships between variables,^30^ thereby informing the process of building causal models.^31^ DAGs are useful for identifying variables which confound the relationship between two variables, thus providing researchers with a set of variables for which adjustment is necessary (or unnecessary) in order to obtain unbiased estimates of the causal relationship between two variables.^32^ We refer to the final (logistic regression) model comprising the outcome (CDF as a binary variable), the primary exposure (total and/or moderate-to-vigorous physical activity as continuous variables) and all identified confounders as the ‘analysis model’ (online supplementary file figure S1—DAG generated by DAGitty software V.2.3, available from www.dagitty.net).^33^

10.1136/archdischild-2017-314138.supp1 Supplementary data

If the analysis model is fitted to a ‘complete case’ dataset, that is, dropping participants who have missing data for any of the variables in the model, SEs will be inflated, and bias may arise. If missingness is dependent only on observed data, that is, data in the analysis model are ‘missing at random’, then multiple imputation can be used to correct this bias. Multiple imputation uses a model based on the analysis model plus auxiliary variables which are selected because (1) they are associated with the missing variables, (2) they are associated with the missingness of the missing variables and (3) they do not have too much missing data (to ensure stable imputation models which therefore produce reliable estimates).

The number of imputations required to achieve convergence of parameter estimates was determined by checking the estimate of the Monte Carlo error (MCE) in relation to the SE of the coefficient being estimated. The number of imputations was increased until MCE reached a value which was <10% of the SE of the estimate. The sample size after imputation was n=13 978, representing children in the ‘core’ ALSPAC sample who were alive at 1 year and who were either a singleton or twin. Multivariable imputation was performed using an imputation sampling method which incorporates all sources of variability and uncertainty in the imputed values.^34^ The analysis model is then fitted to the imputed datasets, using Rubin’s rules to combine the estimates into a single estimate which is unbiased (or less biased) by differential losses to follow-up.^35^ We also used the imputed datasets to estimate (using linear regression) the mean differences in total and moderate-to-vigorous physical activity and sedentary time at age 11 years comparing children who did or did not develop CDF at age 13 or 16 years. This estimated mean difference was adjusted for the same confounders as were included in the analysis model. Although both physical activity variables showed some skewness, these variables were not log transformed to facilitate comparisons with our previously reported analyses.^20 29 36^ All analyses were performed using Stata V.14 (Stata 2015. Stata Statistical Software).

## Results

### Physical activity at age 11 years and CDF at age 13 years

Data to define CDF at age 13 years were more likely to be missing for children born into lower socioeconomic status households and whose mothers were more likely to experience depression and anxiety (online supplementary table S1). Physical activity data at age 11 years were available for 63.6% (42/66) and 67.7% (4504/6654) of children who did or did not have CDF at age 13 years, respectively.

10.1136/archdischild-2017-314138.supp2 Supplementary data

Median levels of total and moderate-to-vigorous physical activity were lower at age 11 years in children who had CDF at age 13 years (table 1). Each 100 cpm increase in mean total physical activity was associated with 25% lower odds of CDF at age 13 years (OR=0.75 (95% CI 0.59 to 0.95)), a 1-percentage point increase (corresponding to approximately 6 min) in the daily proportion of physical activity rated as moderate to vigorous was associated with 16% lower odds of CDF (OR=0.84 (95% CI 0.69 to 1.01)) and an increase of 1 hour per day in sedentary time was associated with 35% higher odds of CDF (OR=1.35 (95% CI 1.02 to 1.79)) (table 2). Children with CDF at age 13 years had lower total (−77.4 (95% CI −136.6 to −18.1) cpm) and moderate-to-vigorous physical activity (−4.83 (95% CI −9.63 to −0.03) min/day), and longer sedentary time (26.3 (95% CI 2.03 to 50.7) min/day) at age 11 years compared with children did not develop CDF (imputed and fully adjusted mean differences).

**Table 1:** Physical activity at age 11 in children who did or did not have chronic disabling fatigue (CDF) at age 13 or 16 years

	Children without CDF at age 13 years (n=4504)*	Children with CDF at age 13 years (n=42)*	P value†
	Median (IQR)	Median (IQR)	
Total physical activity (counts/min)‡	577 (472–710)	482 (409–581)	0.001
Moderate-to-vigorous physical activity (%)§	2.68 (1.62–4.22)	1.77 (1.11–3.42)	0.006
Sedentary time (hours)¶	5.93 (5.09–6.76)	6.55 (6.08–7.35)	0.002

	Children without CDF at age 16 years (n=3854)**	Children with CDF at age 16 years (n=53)**	
Total physical activity (counts/min)‡	573 (468–705)	526 (432–667)	0.08
Moderate-to-vigorous physical activity (%)§	2.65 (1.59–4.17)	2.27 (1.31–3.62)	0.08
Sedentary time (hours)¶	5.98 (5.13–6.80)	6.34 (5.38–6.99)	0.07

*Physical activity data at age 11 years were available for 63.6% (42/66) and 67.7% (4504/6654) of children who did or did not have CDF at age 13 years, respectively.

†Kruskal-Wallis test.

‡Total physical activity=mean counts/min over the 7-day period when the accelerometers were worn.

§Moderate-to-vigorous physical activity=percentage of monitored time when accelerometer recorded ≥3500 counts/min.

¶Sedentary time=mean daily time when accelerometer recorded ≤100 counts/min.

**Physical activity data at age 11 years were available for 72.6% (53/73) and 67.8% (3854/5683) of children who did or did not have CDF at age 16 years, respectively.

**Table 2:** Associations of physical activity at age 11 years with chronic disabling fatigue (CDF) at age 13 years

		Raw data (n=4546)	Imputed data (n=13 978)*
Total physical activity†	Unadjusted OR	0.70 (0.56, 0.88)	0.76 (0.62, 0.93)
	Partially adjusted OR‡	0.68 (0.54, 0.87)	0.75 (0.60, 0.93)
	Fully adjusted OR§	–	0.75 (0.59, 0.95)
	Fully adjusted mean difference (counts/min)§		−77.4 (–136.6, –18.1)
Moderate-to-vigorous physical activity¶	Unadjusted OR	0.79 (0.64, 0.97)	0.84 (0.72, 0.99)
	Partially adjusted OR‡	0.77 (0.62, 0.96)	0.84 (0.70, 0.99)
	Fully adjusted OR§	–	0.84 (0.69, 1.01)
	Fully adjusted mean difference (min/day)§		−4.83 (–9.63, –0.03)
Sedentary time**	Unadjusted OR	1.50 (1.15, 1.95)	1.38 (1.07, 1.79)
	Partially adjusted OR‡	1.50 (1.15, 1.95)	1.39 (1.07, 1.80)
	Fully adjusted OR§	–	1.35 (1.02, 1.79)
	Fully adjusted mean difference (min/day)§		26.3 (2.03, 50.7)

*Variables for the multiple imputation (50 imputations) were as follows: CDF at 13 and 16 years; sex; nighttime sleep duration at 7 and 9 years; body mass index (BMI) at 7 and 9 years; child mood at 9 years (Short Moods and Feelings Questionnaire score); maternal depression at 6 years (Edinburgh Postnatal Depression Scale); maternal anxiety at 6 years (Crown-Crisp Experiential Index); mean test score for English, Mathematics and Science at 11 years (Key Stage 2 tests); maternal life events score (antenatal); self-esteem at 8 years (Global Self Worth subscale from Harter’s Self Perception Profile for Children); Avon Longitudinal Study of Parents and Children family adversity index (antenatal); family adversity index at 8–10 years; life difficulties, hyperactivity and internalising behaviour at 11 years (Strengths and Difficulties Questionnaire); screen time at 6 years; special school arrangements for emotional/behavioural problems at 7 years; conduct problems at 8 years; duration of breastfeeding; family income at 4 years; maternal childhood socioeconomic status; maternal education; maternal age at birth of child; maternal psychopathology at 8–10 years; experienced bullying at 8 years; days spent outdoors at 8 years; poor concentration at school at 7 years; maternal and paternal BMI at 8 years; internalising behaviour at 4 years (Strengths and Difficulties Questionnaire); early puberty; dietary patterns at 3 and 10 years.

†For total physical activity, change is per 100 counts/min increase in mean total physical activity for the whole week.

‡Adjusted for sex and family adversity index (antenatal).

§Adjusted for sex; family adversity index (antenatal); BMI at 7 and 9 years; maternal depression at 6 years; maternal anxiety at 6 years; internalising behaviour at 4 and 11 years; self-esteem at 8 years; child mood at 9 years; early puberty; Key Stage 2 test scores; duration of breastfeeding; family income at 4 years; screen time at 6 years; maternal childhood socioeconomic status; maternal life events score (antenatal); nighttime sleep duration at 8 years; conduct problems at 8 years; dietary patterns at 10 years.

¶For moderate-to-vigorous physical activity, change is per 15 min increase (threshold ≥3500 counts/min).

**For sedentary time, change is per hour increase.

Disabling fatigue of 3–5 months' duration at age 13 years was less strongly associated with total physical activity (raw data unadjusted OR=0.88 (95% CI 0.76 to 1.03); imputed data fully adjusted OR=0.86 (95% CI 0.75 to 0.99)), moderate-to-vigorous physical activity (raw data unadjusted OR=0.94 (95% CI 0.82 to 1.07); imputed data fully adjusted OR=0.90 (95% CI 0.80 to 1.01)) and sedentary time (raw data unadjusted OR=1.22 (95% CI 0.99, to1.51); imputed data fully adjusted OR=1.20 (95% CI 1.00 to 1.44)).

### Physical activity at age 11 years and CDF at age 16 years

Data to define CDF at age 16 years were more likely to be missing for children born into lower socioeconomic status households and whose mothers were more likely to experience depression and anxiety (online supplementary table S2). Physical activity data at age 11 years were available for 72.6% (53/73) and 67.8% (3854/5683) of children who did or did not have CDF at age 16 years, respectively. Median levels of total and moderate-to-vigorous physical activity were lower at age 11 years in children who had CDF at age 16 years, but with weak evidence for these differences (table 1). Total physical activity (OR=0.94 (95% CI 0.77 to 1.15)), moderate-to-vigorous physical activity (OR=0.96 (95% CI 0.82 to 1.13)) and sedentary time (OR=1.16 (95% CI 0.89 to 1.51)) were not associated with CDF at age 16 years (table 3).

**Table 3:** Associations of physical activity at age 11 years with chronic disabling fatigue (CDF) at age 16 years

		Raw data (n=3907)	Imputed data (n=13 978)*
Total physical activity†	Unadjusted OR	0.87 (0.72, 1.04)	0.88 (0.74, 1.05)
	Partially adjusted OR‡	0.91 (0.76, 1.10)	0.91 (0.76, 1.09)
	Fully adjusted OR§	–	0.94 (0.77, 1.15)
	Fully adjusted mean difference (counts/min)§		−14.7 (−70.8, 41.4)
Moderate-to-vigorous physical activity¶	Unadjusted OR	0.89 (0.76, 1.06)	0.90 (0.78, 1.04)
	Partially adjusted OR‡	0.95 (0.80, 1.13)	0.94 (0.81, 1.09)
	Fully adjusted OR§	–	0.96 (0.82, 1.13)
	Fully adjusted mean difference (min/day)§		−0.82 (−5.48, 3.85)
Sedentary time**	Unadjusted OR	1.20 (0.94, 1.52)	1.23 (0.97, 1.56)
	Partially adjusted OR‡	1.17 (0.92, 1.49)	1.22 (0.96, 1.54)
	Fully adjusted OR§	–	1.16 (0.89, 1.51)
	Fully adjusted mean difference (min/day)§		12.6 (−9.76, 35.0)

*Variables for the multiple imputation (50 imputations) were as follows: CDF at 13 and 16 years; sex; nighttime sleep duration at 7 and 9 years; body mass index (BMI) at 7 and 9 years; child mood at 9 years (Short Moods and Feelings Questionnaire score); maternal depression at 6 years (Edinburgh Postnatal Depression Scale); maternal anxiety at 6 years (Crown-Crisp Experiential Index); mean test score for English, Mathematics and Science at 11 years (Key Stage 2 tests); maternal life events score (antenatal); self-esteem at 8 years (Global Self Worth subscale from Harter’s Self Perception Profile for Children); Avon Longitudinal Study of Parents and Children family adversity index (antenatal); family adversity index at 8–10 years; life difficulties, hyperactivity and internalising behaviour at 11 years (Strengths and Difficulties Questionnaire); screen time at 6 years; special school arrangements for emotional/behavioural problems at 7 years; conduct problems at 8 years; duration of breastfeeding; family income at 4 years; maternal childhood socioeconomic status; maternal education; maternal age at birth of child; maternal psychopathology at 8–10 years; experienced bullying at 8 years; days spent outdoors at 8 years; poor concentration at school at 7 years; maternal and paternal BMI at 8 years; internalising behaviour at 4 years (Strengths and Difficulties Questionnaire); early puberty; dietary patterns at 3  and 10 years.

†For total physical activity, change is per 100 counts/min increase in mean total physical activity for the whole week

‡Adjusted for sex and family adversity index (antenatal).

§Adjusted for sex; family adversity index (antenatal); BMI at 7 and 9 years; maternal depression at 6 years; maternal anxiety at 6 years; internalising behaviour at 4 and 11 years; self-esteem at 8 years; child mood at 9 years; early puberty; Key Stage 2 test scores; duration of breastfeeding; family income at 4 years; screen time at 6 years; maternal childhood socioeconomic status; maternal life events score (antenatal); nighttime sleep duration at 8 years; conduct problems at 8 years; dietary patterns at 10 years.

¶For moderate-to-vigorous physical activity, change is per 15 min increase (threshold ≥3500 counts/min).

**For sedentary time, change is per hour increase.

## Discussion

Children who had CDF at age 13 years had lower levels of physical activity at age 11 years. For each additional 100 cpm of total physical activity, the odds of CDF at age 13 years were reduced by 25%. Given that children with CDF in this cohort had a median physical activity level of 482 cpm, an additional 100 cpm represents a 20% increase in the overall amount of time spent moving around. For each additional 1% of monitored time spent in moderate-to-vigorous activity, the odds of CDF were reduced by 16%. A percentage point of moderate-to-vigorous physical activity represents approximately 8 min of an activity such as walking or playing football (based on the average accelerometer wear time of 774 min, hence 1% of 774=7.7 min). Each additional hour of sedentary time per day was associated with 35% higher odds of CDF. Disabling fatigue of only 3–5 months’ duration at age 13 years had weaker associations with physical activity, and CDF at age 16 years was not associated with physical activity at age 11 years. This suggests that the association of physical activity at age 11 years with CDF (of >6 months’ duration) at age 13 years may reflect reverse causality, that is, the lower levels of physical activity at age 11 years are a consequence of chronic fatigue which is already present or developing and which persists until the child is 13 years old.

### Strengths and limitations

Our study is the first to investigate a temporal relationship between levels of physical activity and chronic disabling fatigue (as a proxy for CFS/ME) in early and late adolescence. The main strengths of our study are that it used prospectively collected data from a large population based birth cohort, and that we used objective measures of physical activity. The use of DAGs enabled us to examine temporal relationships and to adjust for multiple potential confounders in a systematic way which takes account of the previous evidence in the field.^30^ Furthermore, we used multiple imputation to adjust for higher losses to follow-up that occur among cohort participants from less affluent social groups.^21^

The main limitation of our study is that children were not assessed by a doctor, which is why we describe our outcome as ‘chronic disabling fatigue’, a proxy for CFS/ME. At age 13 years, our classification was based only on parental report of fatigue, with limited data on degree of disability.^6^ We may have excluded teenagers whose parents under-reported the impact of fatigue and/or included teenagers whose parents overestimated fatigue or its impact. However, our CDF outcome at age 16 years combined parental data with a child-completed Chalder Fatigue Questionnaire (CFQ) score.^7^ At this age, we classified children as not having CDF if they had a CFQ score <19, a threshold which has high sensitivity and specificity for CFS/ME in adults.^23^ The threshold used to define minutes of moderate-to-vigorous physical activity from the accelerometer data affects estimates of physical activity. To facilitate comparisons with the other ALSPAC papers, we have used the threshold of 3500 cpm.^25^ We recognise that others have suggested that the Evenson *et al* 37 cut-point of ≥2296 cpm may have wider validity and reliability in the target group, particularly for population-based samples.^38^

We cannot be sure to what extent the association of physical activity at age 11 years with CDF at age 13 years reflects reverse causation. In a UK paediatric CFS/ME clinical cohort, the median (IQR) self-reported duration of illness for children aged under 12 years was 12 (7–23) months and in children aged 12–18 years it was 18 (11–28) months.^3^ Furthermore, our previous analysis of CDF among children in the ALSPAC cohort at age 13, 16 and 18 years showed that 75% of children recovered after 2–3 years.^10^ These two studies suggest that the proportion of children in this study whose chronic fatigue would be longer than 2 years’ duration would be low, and that any association of physical activity at age 11 years with CDF at age 13 years would not reflect reverse causation. However, analysis of electronic primary care data in the UK showed that children with CFS/ME had substantially higher healthcare needs than matched population controls for at least 10 years before their diagnosis.^39^ This suggests that parents have underestimated the duration of their child’s fatigue, or that paediatric CFS/ME begins in a mild form which develops into more severe CFS/ME. This is plausible if the milder form of CFS/ME does not lead to notable absences from school or college.

### Our findings in the context of the studies

Our results are not consistent with the only other study to investigate a possible association between physical activity and CFS/ME in adolescents (age 12–18 years), which found no differences in levels of physical activity 12 months before infectious mononucleosis comparing those who did or did not subsequently develop CFS/ME.^19^ However, this was based on self-report and a small sample (39 cases and 39 controls), and there was tentative evidence that adolescents who developed CFS/ME had lower scores for vigorous exercise (P=0.09) and were spending more time napping (P=0.08) in the year before infectious mononucleosis. Our results are consistent with a population-based study which reported 60% higher odds (OR=1.6 (95% CI 1.1 to 2.3)) of ‘severe fatigue’ in adolescents age 13–16 years among those who had reported >4 hours per day of sedentary time (compared with <2 hours/day) 2 years previously.^40^ In contrast with our findings, they also reported an increased risk (50% higher odds, OR=1.5 (1.2 to 2.1)) associated with being physically active (exercising >2 times each week for an hour or more). The exposure and outcome measures in this study were self-reported (WHO Health Behaviour in School-aged Children questionnaire), and ‘severe fatigue’ was defined as extreme tiredness occurring more than once per week (in the last month). Crucially, this definition does not exclude tiredness caused by playing sport, which might explain the positive association between physical activity and risk of severe fatigue.

## Conclusion

Our results suggest a possible association between lower levels of physical activity and the development of chronic fatigue in younger teenagers, but this could be explained by reverse causation. Our findings need to be replicated in a larger sample using accelerometer and fatigue data collected at repeated time points further back in time from the outcome, such that the true relationship between physical activity levels and onset of chronic fatigue can be determined.
