# Supplementary figures and images for: Physical activity at age 11 years and chronic disabling fatigue at ages 13 and 16 years in a UK birth cohort
Source: Arch Dis Child. 2018 Jan 30;103(6):586–91. doi: 10.1136/archdischild-2017-314138 (PMC5965358; doi:10.1136/archdischild-2017-314138)

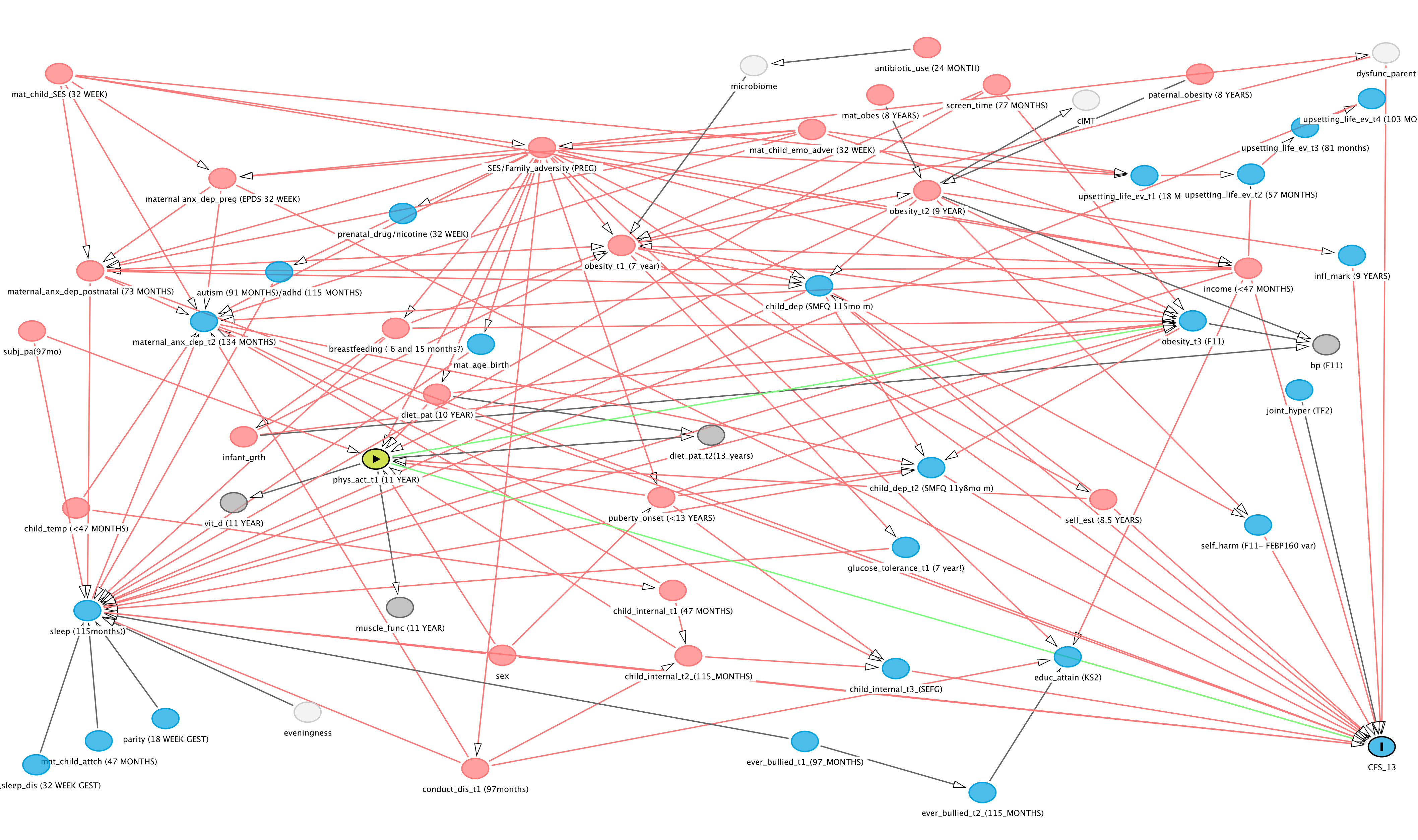

Supplement: Supplementary data [file archdischild-2017-314138supp001.pdf]
