# Supplementary material for: Physical activity at age 11 years and chronic disabling fatigue at ages 13 and 16 years in a UK birth cohort
Source: Arch Dis Child. 2018 Jan 30;103(6):586–91. doi: 10.1136/archdischild-2017-314138 (PMC5965358; doi:10.1136/archdischild-2017-314138)
Supplement: Supplementary data [file archdischild-2017-314138supp002.docx]

**Table S1: Cohort characteristics for children classified with or without chronic disabling fatigue (CDF), or without data for this outcome, at age 13 years**

|  |  | Children without CDF at age 13 years (n=6654) | Children with CDF at age 13 years (n=66) | P-value^*^ | Children missing outcome data (n=7258) | P-value^**^ |
| --- | --- | --- | --- | --- | --- | --- |
| Maternal age (at birth of child) | median (IQR) | 29 (26 - 32) | 31 (28 - 33) | 0.01 | 27 (23 - 30) | <0.001 |
| Maternal education | CSE/Vocational | 1371 (21.2%) | 13 (20.3%) | 0.10 | 2344 (39.9%) | <0.001 |
|  | O-level | 2270 (35.1%) | 16 (25.0%) |  | 2010 (34.2%) |  |
|  | A-level | 1722 (26.6%) | 17 (26.6%) |  | 1055 (18.0%) |  |
|  | Degree | 1112 (17.2%) | 18 (28.1%) |  | 470 (8.0%) |  |
| Maternal EPDS score^***^ | median (IQR) | 5 (2 - 9) | 8 (5 - 12) | 0.001 | 6 (2 - 10) | <0.001 |
| Maternal CCEI score^***^ | median (IQR) | 4 (2 - 7) | 6 (4 - 10) | <0.001 | 5 (2 - 7) | 0.03 |
| Sex of child | Female, n (%) | 3313 (49.8%) | 35 (53.0%) | 0.60 | 3410 (47.0%) | 0.001 |
| SDQ total score (range 0 - 40)^****^ | median (IQR) | 5 (3 - 9), n=5812 | 10 (5 - 16) | <0.001 | 6 (4 - 10), n=1112 | <0.001 |

* Chi-squared test for proportions, Kruskal-Wallis test for medians, comparing characteristics between children with or without CDF at age 13

** Chi-squared test for proportions, Kruskal-Wallis test for medians, comparing characteristics between children with or without outcome data

*** Edinburgh Postnatal Depression Scale (EPDS) or Crown-Crisp Experiential Index (CCEI) score when child was 6 years old

**** Strengths & Difficulties Questionnaire (SDQ) score: life difficulties were quantified by means of the SDQ, which was completed by parents as part of the “Being a Boy/Girl At 140 Months” questionnaire. The SDQ is a behavioural screening questionnaire designed to assess 25 attributes in children up to 16 years old. The SDQ comprises five 5-item subscales (emotional symptoms, conduct problems, hyperactivity inattention, peer relationships problems, prosocial behaviour). A ‘total difficulties’ score is calculated by adding scores for the first four subscales (excluding prosocial behaviour), yielding a total score with a range of 0 - 40. The SDQ is a widely used, valid and reliable screening questionnaire for mood disorders in children.

**Table S2: Cohort characteristics for children classified with or without chronic disabling fatigue (CDF), or without data for this outcome, at age 16 years**

|  |  | Children without CDF at age 16 years (n=5683) | Children with CDF at age 16 years (n=73) | P-value^*^ | Children missing outcome data (n=8222) | P-value^**^ |
| --- | --- | --- | --- | --- | --- | --- |
| Maternal age (at birth of child) | median (IQR) | 29 (26 - 32) | 31 (28 - 34) | 0.02 | 27 (24 - 30) | <0.001 |
| Maternal education | CSE/Vocational | 1064 (19.3%) | 8 (11.3%) | 0.06 | 2656 (32.3%) | <0.001 |
|  | O-level | 1895 (34.4%) | 23 (32.4%) |  | 2378 (28.9%) |  |
|  | A-level | 1553 (28.2%) | 19 (26.8%) |  | 1222 (14.9%) |  |
|  | Degree | 1005 (18.2%) | 21 (29.6%) |  | 574 (7.0%) |  |
| Maternal EPDS score^***^ | median (IQR) | 5 (2 - 9) | 7 (4 - 11) | 0.008 | 6 (2 - 10) | <0.001 |
| Maternal CCEI score^***^ | median (IQR) | 4 (2 - 7) | 5 (4 - 9) | 0.002 | 5 (2 - 7) | <0.001 |
| Sex of child | Female, n (%) | 2965 (52.2%) | 49 (67.1%) | 0.01 | 3744 (45.5%) | <0.001 |
| SDQ total score (range 0 - 40)^****^ | median (IQR) | 5 (3 - 8), n=4823 | 6 (4 - 11), n=62 | 0.007 | 6 (4 - 10), n=2105 | <0.001 |

* Chi-squared test for proportions, Kruskal-Wallis test for medians, comparing characteristics between children with or without CDF at age 16

** Chi-squared test for proportions, Kruskal-Wallis test for medians, comparing characteristics between children with or without outcome data

*** Edinburgh Postnatal Depression Scale (EPDS) or Crown-Crisp Experiential Index (CCEI) score when child was 6 years old

**** Strengths & Difficulties Questionnaire (SDQ) score: life difficulties were quantified by means of the SDQ, which was completed by parents as part of the “Being a Boy/Girl At 140 Months” questionnaire. The SDQ is a behavioural screening questionnaire designed to assess 25 attributes in children up to 16 years old. The SDQ comprises five 5-item subscales (emotional symptoms, conduct problems, hyperactivity inattention, peer relationships problems, prosocial behaviour). A ‘total difficulties’ score is calculated by adding scores for the first four subscales (excluding prosocial behaviour), yielding a total score with a range of 0 - 40. The SDQ is a widely used, valid and reliable screening questionnaire for mood disorders in children.
